# Leveraging a High-Throughput Screening Method to Identify Mechanisms of Individual Susceptibility Differences in a Genetically Diverse Zebrafish Model

**DOI:** 10.3389/ftox.2022.846221

**Published:** 2022-04-29

**Authors:** Dylan J. Wallis, Jane La Du, Preethi Thunga, Daniel Elson, Lisa Truong, Siva K. Kolluri, Robyn L. Tanguay, David M. Reif

**Affiliations:** ^1^ Bioinformatics Research Center, Department of Biological Sciences, North Carolina State University, Raleigh, NC, United States; ^2^ Sinnhuber Aquatic Research Laboratory, Department of Environmental and Molecular Toxicology, Oregon State University, Corvallis, OR, United States; ^3^ Cancer Research Laboratory, Department of Environmental and Molecular Toxicology, Oregon State University, Corvallis, OR, United States

**Keywords:** high-throughput (HT) approaches, zebrafish, computational toxicology, mechanism, genomic, abamectin, pathways

## Abstract

Understanding the mechanisms behind chemical susceptibility differences is key to protecting sensitive populations. However, elucidating gene-environment interactions (GxE) presents a daunting challenge. While mammalian models have proven useful, problems with scalability to an enormous chemical exposome and clinical translation faced by all models remain; therefore, alternatives are needed. Zebrafish (*Danio rerio*) have emerged as an excellent model for investigating GxE. This study used a combined bioinformatic and experimental approach to probe the mechanisms underlying chemical susceptibility differences in a genetically diverse zebrafish population. Starting from high-throughput screening (HTS) data, a genome-wide association study (GWAS) using embryonic fish exposed to 0.6 μM Abamectin revealed significantly different effects between individuals. A hypervariable region with two distinct alleles–one with G at the SNP locus (GG) and one with a T and the 16 bp deletion (TT)–associated with differential susceptibility was found. Sensitive fish had significantly lower *sox7* expression. Due to their location and the observed expression differences, we hypothesized that these sequences differentially regulate *sox7.* A luciferase reporter gene assay was used to test if these sequences, alone, could lead to expression differences. The TT allele showed significantly lower expression than the GG allele in MCF-7 cells. To better understand the mechanism behind these expression differences, predicted transcription factor binding differences between individuals were compared *in silico*, and several putative binding differences were identified. EMSA was used to test for binding differences in whole embryo protein lysate to investigate these TF binding predictions. We confirmed that the GG sequence is bound to protein in zebrafish. Through a competition EMSA using an untagged oligo titration, we confirmed that the GG oligo had a higher binding affinity than the TT oligo, explaining the observed expression differences. This study identified differential susceptibility to chemical exposure in a genetically diverse population, then identified a plausible mechanism behind those differences from a genetic to molecular level. Thus, an HTS-compatible zebrafish model is valuable and adaptable in identifying GxE mechanisms behind susceptibility differences to chemical exposure.

## 1 Introduction

Population-wide differences in susceptibility to chemical exposures exert significant impacts in medicine and risk assessment (Suter et al., 2004; Roden et al., 2011; Gamazon and Perera, 2012; Motsinger-Reif et al., 2013). Identifying compounds that have vast differences in individual susceptibility across a genetically diverse population has important implications for how we prescribe drugs to treat disease, deal with environmental exposures, and regulate chemical producers (Suter et al., 2004; Qin et al., 2016). As of 2013 there were over 80,000 chemicals registered with the EPA that people could be exposed to (United States, 1986; Bergeson, 2000; Kinch et al., 2014). Yet, the mechanisms that lead to population-level variation in susceptibility remain poorly understood (Zhou et al., 2017; Jerry et al., 2018).

The National Research Council (NRC) stated in their document on the future of risk assessment, “Science and Decisions”, that susceptibility differences across different chemicals required more attention (National Research Council, 2009). In 2016, the Frank R. Lautenberg act amended The Toxic Substances Control Act (TSCA) to include the consideration of susceptible populations in risk assessment (United States, 1986; Mortensen and Euling, 2013; Mortensen et al., 2018). This cemented the importance of understanding the variables that can lead to differences in susceptibility in a population to diseases caused by chemical exposure.

Unfortunately, the study of how genetic differences and gene-environment interactions (GxE) can affect (sub)population response to an exposure is challenging for several reasons: Sample sizes of sufficient power are hard to achieve; Mechanisms are hard to parse out, Environmental variables are hard to control and separate; and Relating an exposure to a specific phenotype is challenging (Le Marchand and Wilkens, 2008; Freedman et al., 2011; McAllister et al., 2017; Zhou et al., 2017). Current knowledge says that regulatory variation in genes may be the primary cause of phenotypic differences in humans (Mortensen and Euling, 2013). These regions are harder to disentangle to gain an understanding of their effect on susceptibility. In addition to this, while new approach methodologies (NAMs) can identify chemicals with the potential to elicit clinically observable adverse effects, comprehensive risk assessment is hampered by differential susceptibility across exposed individuals. In past studies, animal models have been used to identify and implicate specific genes that lead to susceptibility to different diseases in humans (Mortensen and Euling, 2013; Mortensen et al., 2018). Current risk assessment methods use uncertainty factors when calculating acceptable exposure levels to approximate differences between susceptible and resistant populations (Mortensen and Euling, 2013; Mortensen et al., 2018).

Zebrafish (*Danio rerio*) have emerged as an excellent model of human disease that are well suited for high-throughput *in vivo* assays: They produce a large number of offspring; They have phenotypes that can easily be compared to human diseases; Their embryos are transparent and develop *ex vivo*; Phenotypes can be assessed as early as 24 h after fertilization; And they can be separated into wells to be exposed individually to allow for a highly controlled environment (Truong et al., 2011; Truong et al., 2014; Garcia et al., 2016; Truong et al., 2016; Balik-Meisner et al., 2018a). Chemical testing using zebrafish provides phenotypic responses that can reveal the etiology of many adverse outcomes (Truong et al., 2011; Truong et al., 2014; Garcia et al., 2016; Reif et al., 2016; Truong et al., 2016; Zhang et al., 2017; Balik-Meisner et al., 2018a; Garcia et al., 2018). Zebrafish are also the only vertebrate model that can be assayed in a high throughput manner at the phylotypic stage of development that is most similar to vertebrate early development (Garcia et al., 2016). Unlike an *in vitro* system or another *in vivo* vertebrate system, zebrafish can reveal the mechanisms of complex phenotypic responses to chemicals in a high-throughput and cost-effective manner (Truong et al., 2011; Truong et al., 2014; Garcia et al., 2016; Truong et al., 2016; Balik-Meisner et al., 2018a). The T5D zebrafish line used here is an outbred zebrafish line that mimics human genetic diversity and acts as an excellent proxy for assessing differential susceptibility to many different chemicals (Balik-Meisner et al., 2018a; Balik-Meisner et al., 2018b). A study on the genetic diversity of T5D zebrafish found 10.3–20.1 M SNPs and 2.8–5.6 M indels across the population (Balik-Meisner et al., 2018a; Balik-Meisner et al., 2018b). As a comparison, the 1,000 genome project found that humans had 84.7 M SNP, 3.6 M short indels (The 1000 Genomes Project Consortium et al., 2015).

This study acts as a proof of concept that HTS for developmental effects of exposure to a large array of chemicals can identify those associated with GxE that elicit differences in individual susceptibility across the genetically diverse T5D zebrafish line (Truong et al., 2011; Truong et al., 2014; Truong et al., 2016; Balik-Meisner et al., 2018a). The data in this study uses information gained from a screening of 1078 EPA ToxCast phase 1 and 2 chemicals (Truong et al., 2011; Truong et al., 2014; Truong et al., 2016; Balik-Meisner et al., 2018a). Several chemicals, including abamectin, showed substantial differences in developmental effects across zebrafish, suggesting that underlying genetic variation may play a role in exposure-response. Abamectin, an agricultural insecticide that has been associated with neurotoxicity, developmental effects, and endocrine disruption according to a risk assessment by the European Food Safety Authority (Anastassiadou et al., 2020), was selected for further investigation due to robust population susceptibility differences across multiple rounds of rangefinder experiments. In a genome-wide association study (GWAS) of developmental exposure to Abamectin, multiple single-nucleotide polymorphisms (SNPs) were discovered that were associated with differential effects (Balik-Meisner et al., 2018a). Some of these SNPs were located upstream of the SRY-Box Transcription Factor 7 (*sox7*) gene. Human SOX7 plays several important roles in development including cell differentiation, regulation between cell proliferation and differentiation, lineage determination, regulation of angiogenesis and vasculogenesis, and also acts as a tumor suppressor (Stovall et al., 2014). Zebrafish Sox7 is orthologous to human SOX7 and is predicted to play a similar role in producing a DNA-binding transcription factor that helps to guide development (Zfin Gene, 2021).

**FIGURE 1:**
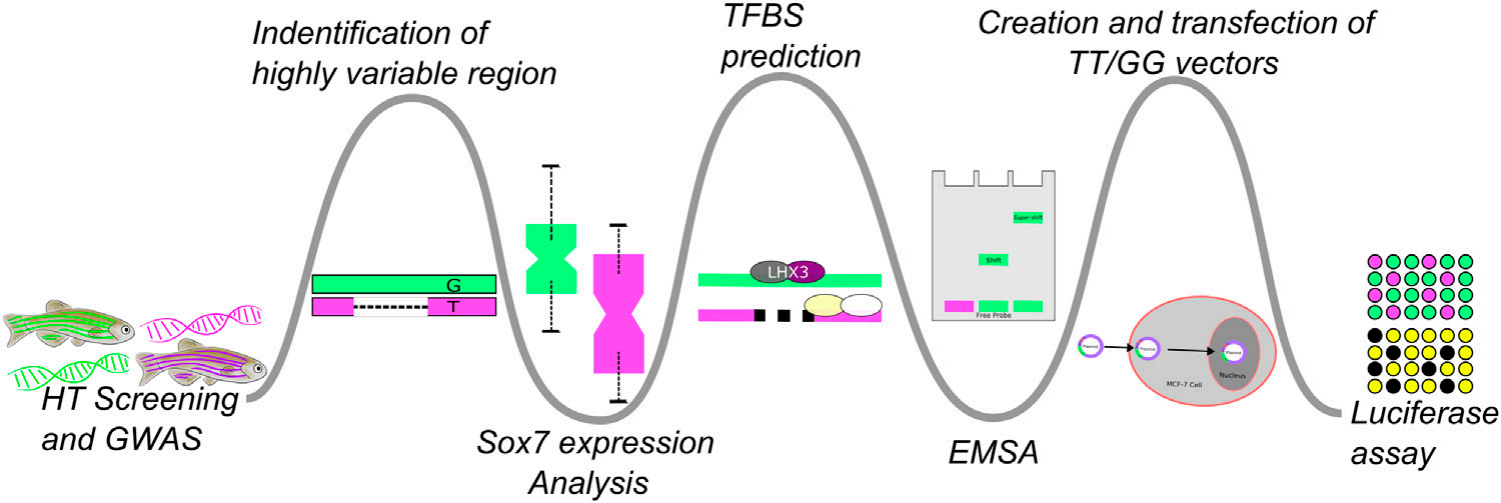
This figure illustrates the experimental flow of this study which could be tailored or branched, based on the progression of biochemical and wet-lab experiments, to apply it to similar studies. Bioinformatic approaches were used to generate hypotheses which were then tested *via* biochemical experimentation. A high-throughput GWAS experiment followed by a more *in-depth* analysis of a single genetic region was followed-up with RT-PCR to test if *sox7* expression differed in the affected an unaffected fish. This was then followed up by *in silico* analysis of the region to identify possible TF binding differences. The results of that comparison were confirmed with EMSA to test for differences in binding affinities across the variants and their ability to cause differential expression was probed with a luciferase assay.

When interrogated using real-time polymerase chain reaction (real-time PCR) *sox7* exhibited significant expression differences between fish that were sensitive to abamectin expression (“affected”) and fish that were not (“unaffected”) (Balik-Meisner et al., 2018a). This led to the hypothesis that susceptibility to abamectin was affected by regulation of *sox7.* Here, we describe a discovery path (Figure 1) of bioinformatically-guided experimentation to test our hypothesis that genetic variation influences population responses to environmental exposure (GxE). As part of the broader approach, this study aimed to validate a plausible mechanism behind HTS-scale patterns of differential response down to a detailed molecular and genetic level.

## 2 Materials and Methods

### 2.1 Experimental Overview

A high-throughput screening (HTS), genome-wide association study (GWAS) was performed to identify loci that were associated with individual differences in susceptibility across an outbred zebrafish population as described in Balik-Meisner et al. (2018a). Briefly, the study leveraged data from thousands of chemicals, as well as a method that quantified morphological effects of developmental exposure in an outbred zebrafish population to identify chemicals that showed high variability in effect across the population (Zhang et al., 2017; Balik-Meisner et al., 2018a). Abamectin was chosen from these high-variance chemicals. After identifying a critical concentration, larval zebrafish were exposed (Balik-Meisner et al., 2018a). Affected and unaffected zebrafish larvae were collected and their DNA was individually sequenced to identify loci associated with susceptibility (Balik-Meisner et al., 2018a). This study identified significant expression differences in *sox7* between affected and unaffected zebrafish using PCR (Balik-Meisner et al., 2018a). A combined bioinformatic and biochemical approach was used to validate the results from the previous study and identify the mechanism that leads to susceptibility differences. Transcription factor binding site (TFBS) prediction was used to identify putative transcription factor binding differences between individual fish. Following the TFBS prediction analysis, an electrophoretic mobility shift assay (EMSA) was used to assess binding between these sequences and proteins in zebrafish nuclear protein extract. A luciferase assay was then used to measure expression differences between the two presumed regulatory regions.

### 2.2 Zebrafish Model

Tropical 5D (T5D) wild type zebrafish were housed at Sinnhuber Aquatic Research Laboratory at Oregon State University, in a density of approximately 1,000 fish per 100-gallon tank. Tank water consisting of reverse osmosis water supplemented with Instant Ocean™ salts were kept at standard laboratory conditions of 28°C on a 14-h light/10-h dark photoperiod. The water was at a salinity of 600 μS and maintained at a pH of 7.4 (Mandrell et al., 2012; Barton et al., 2016). Mass spawning funnels were placed into the tanks the night prior, and the following morning embryos were collected, staged and cleaned (Kimmel et al., 1995). T5D zebrafish originated from 25 small group crosses containing three males and three females imported from a breeding facility. Following generations have been bred with equal proportions of offspring from a minimum of 25 small group crosses, each group containing three males and up to three females (Balik-Meisner et al., 2018b). For more details on the genetic diversity of the T5D line please see Balik-Meisner et al. (2018b). Zebrafish embryos were obtained *via* group spawning and were dechorionated after collection and placed in 96 well plates as detailed in multiple papers by Truong et al. (2011) and Truong et al. (2014). The T5D zebrafish line was used for all zebrafish experiments in this study.

### 2.3 Deep Reanalysis

The preliminary study identified a putative hyper-variable region upstream of *sox7* that was associated with susceptibility on chromosome 20. In order to identify loci that were associated with susceptibility within this region a secondary analysis of chromosome 20 was done. Paired end reads from the previous HT sequencing were trimmed with trimmomatic version 0.39 using the following parameters: ILLUMINACLIP:TruSeq3-PE.fa:2:30:10, LEADING:3, TRAILING:3, SLIDINGWINDOW:4:15, and MINLEN:36 (Bolger et al., 2014). Quality control was done on the sequences using fastqc to ensure the quality of the sequences for further analysis (Ewels et al., 2016). Sequences were aligned using the software package Burrows-Wheeler Aligner (BWA-MEM) with standard parameters (bwa.1, 2021).

Sam files were converted to bam files using the software package samtools (samtools). The bam files were sorted, indexed, and duplicates were marked using the software package picard prior to analysis (Picard Tools - By Broad Institute, 2021). Joint genotyping was done using GenomeAnalysisToolkit (GATK) HaplotypeCaller and gvcf files were merged using GATK CombineGVCFs (McKenna et al., 2010; DePristo et al., 2011). After variants were called, GATK VariantFiltration was used to filter out SNPs with the parameters: QualByDepth (QD) < 2, FisherStrand (FS) > 60, RMSMappingQuality (MQ) < 40, MQRankSum < −12.5, and ReadPosRankSum < −8; as well as indels with QD < 2, FS > 200.0, and ReadPosRankSum < −20 (DePristo et al., 2011). Finally, association testing was done in PLINK version 1.9 using Fisher’s exact test with a Bonferroni correction to correct for multiple testing (Purcell et al., 2007).

### 2.4 Transcription Factor Binding Site Prediction

#### 2.4.1 Software

MatInspector, an online program, was used to analyze the sequences upstream of *sox7* for each individual fish. MatInspector uses information on core sequences and nucleotide data on a number of transcription factors to generate a list of putative transcription factor binding sites, scored for goodness of fit using a scoring algorithm. For a more *in-depth* explanation of MatInspector see Cartharius et al. (2005) or the Genomatix website (Cartharius et al., 2005). A core similarity score of 0.75 was used to filter out unlikely matches.

#### 2.4.2 Binding Site Curation

Differences between sequences with G or T at the primary SNP loci were compared using a chi-squared test to identify significant differences using the observed number of each predicted binding site across both genotypes. The test was done using the chisq.test command from the R package stats version 3.6.3 (R Core Team, 2020). This list of putative TFBSs were then curated manually by selecting transcription factors with homologues in zebrafish and sites that were within the 44 bp area between the beginning of the Indel and the SNP.

### 2.5 Luciferase Assay

#### 2.5.1 Assay

Double stranded oligos corresponding to the region −900 to +29 bp of *Sox7*-201 variant TSS were synthesized and cloned in to pUCIDT vectors by IDT. Additional recognition sequence for restriction enzymes NheI and XhoI were added to ends for cloning into the minimal promoter pGL4.23 [*luc2/minP*] vector (Promega, E8411) for the luciferase assay. Sequences from positive clones were verified by Sanger sequencing at the Oregon State University Center for Genome Research and Biocomputing Core. Plasmids were purified with ZymoPURE Plasmid Miniprep Kit (Zymo Research).

MCF7 cells were seeded in a 24-well plate in 10% serum Dulbecco’s Modified Eagle Medium (DMEM). The cells were transfected the following morning with the TT sequence, GG sequence, or vector control reporters as well as a β-galactose (β-gal) plasmid using 300 ng reporter, 200 ng β-gal, and 2 μL lipofectamine per well. The sequences inserted into the vector are given below, annotated as: restriction enzyme sites underlined; transcription start site (TSS) in bold.

GG sequence: GCT​AGCACA​TAA​ATC​CAA​TTC​ATT​TAG​CGA​AAA​TAT​TTA​TTT​TTA​CGA​TAT​TTA​TGA​ATT​GCG​AAT​TGT​GTG​TTG​TAT​TTA​TGA​GTT​GTG​TTT​TGC​ATT​TAC​AAT​TTG​GAT​TTT​GTA​AAT​TTT​GAG​TTG​TGC​ATT​AAT​TTT​ATT​TTG​TAA​ATG​TAT​TAT​TTT​TGA​GAC​TGA​TCT​GGC​TCC​ATA​AAA​ATC​CTG​GTT​TTT​ATT​TGT​AGT​ATT​CCT​AGA​AAG​CTT​ATT​GTA​TAT​TTC​TAG​TCA​AGC​TTG​AAA​ATA​AAT​GTT​AAA​ATA​TGG​GTG​AAA​TAC​GGA​ACA​AAA​TAT​AGA​TCA​TGC​TAA​ATA​TAT​ATA​TAT​ATA​TGT​ATA​TAT​TTG​TGC​TTT​AAA​TGA​TTG​CAC​ATT​TAC​AAT​CTT​AGA​AAG​ACA​GTA​AAA​GGT​ATA​AAT​AAT​AAA​ATT​ACA​ATG​AAT​TAG​TAT​TTT​GGA​TTT​TTT​TTT​TTT​TTT​TGC​AAA​AAA​TGC​TTC​TCC​TTT​CTG​GTC​ACT​ACA​GAT​ACA​TTT​GCT​TTC​ACA​TAA​AAT​AAA​ACA​CAT​AGT​CAT​ATC​CCT​TAT​CAA​ATG​TGC​ATT​ACT​GAA​ACT​GAA​AAA​CAC​GTC​TTT​TAT​TTG​TTT​GAT​ATT​GTG​GCA​AAA​CTT​GAG​GTC​TCA​ATG​CAA​ATC​ATA​GAT​CCC​AAT​AAC​ACC​AAC​ATT​CAC​CTG​TAA​ACT​TTT​AGC​ATT​ACA​TTA​TAT​TGC​TGT​TTA​ATT​CAC​ACA​GCG​CTC​ACA​AAA​CCA​CTC​GCA​CGC​CTG​CAT​TTC​CCC​TAT​GCT​AAT​CAG​ACG​AAA​TAG​GAA​GAA​GTG​CCT​CCG​AAT​GCA​GAA​CCA​CAA​AGA​TGT​TTA​TCT​CGC​TTT​CGG​CAC​TTC​ACA​CCC​AAA​AAT​AAT​GTG​CAG​GTA​GGA​CCA​GGA​GAG​CAG​GAA​GTG​GAC​TGT​CAT​TTT​TTG​TCG​GAA​TGT​TTT​TTT​GCA​ATC​TGG​GGC​ATC​AAG​CTC​CTC​TTC​CTG​TTC​CTG​GAG​GAA​TAA​AGC​CCA​CAT​TTT**AAT​GAT​GCT​CAT​CCT​CTG​CTT​GTT​CAC​TGCTC​GAG**.

TT sequence: GCT​AGC​ACAT​AAA​TCC​AAT​TCA​TTT​AGC​GAA​AAT​ATT​TAT​TTT​TAC​GAT​ATT​TAT​GAA​TTG​CGA​ATT​GTG​TGT​TGT​ATT​TAT​GAG​TTG​TGT​TTT​GCA​TTT​ACA​ATT​TGG​ATT​TTG​TAA​ATT​TTG​AGT​TGT​GCA​TTA​ATT​TTA​TTT​TGT​AAA​TGT​ATT​ATT​TTT​GAG​ACT​GAT​CTG​GCT​CCA​TAA​AAA​TCC​TGG​TTT​TTA​TTT​GTA​GTA​TTC​CTA​GAA​AGC​TTA​TTG​TAT​ATT​TCT​AGT​CAA​GCT​TGA​AAA​TAA​ATG​TTA​AAA​TAT​GGG​TGA​AAT​ACG​GAA​CAA​AAT​ATA​GAT​CAT​GCT​AAA**|**TAT​ATA​TTT​GTG​CTT​TAA​ATT​ATT​GCA​CAT​TTA​CAA​TCT​TAG​AAA​GAC​AGT​AAA​AGG​TAT​AAA​TAA​TAA​AAT​TAC​AAT​GAA​TTA​GTA​TTT​TGG​ATT​TTT​TTT​TTT​TTT​TTG​CAA​AAA​ATG​CTT​CTC​CTT​TCT​GGT​CAC​TAC​AGA​TAC​ATT​TGC​TTT​CAC​ATA​AAA​TAA​AAC​ACA​TAG​TCA​TAT​CCC​TTA​TCA​AAT​GTG​CAT​TAC​TGA​AAC​TGA​AAA​ACA​CGT​CTT​TTA​TTT​GTT​TGA​TAT​TGT​GGC​AAA​ACT​TGA​GGT​CTC​AAT​GCA​AAT​CAT​AGA​TCC​CAA​TAA​CAC​CAA​CAT​TCA​CCT​GTA​AAC​TTT​TAG​CAT​TAC​ATT​ATA​TTG​CTG​TTT​AAT​TCA​CAC​AGC​GCT​CAC​AAA​ACC​ACT​CGC​ACG​CCT​GCA​TTT​CCC​CTA​TGC​TAA​TCA​GAC​GAA​ATA​GGA​AGA​AGT​GCC​TCC​GAA​TGC​AGA​ACC​ACA​AAG​ATG​TTT​ATC​TCG​CTT​TCG​GCA​CTT​CAC​ACC​CAA​AAA​TAA​TGT​GCA​GGT​AGG​ACC​AGG​AGA​GCA​GGA​AGT​GGA​CTG​TCA​TTT​TTT​GTC​GGA​ATG​TTT​TTT​TGC​AAT​CTG​GGG​CAT​CAA​GCT​CCT​CTT​CCT​GTT​CCT​GGA​GGA​ATA​AAG​CCC​ACA​TTT​T**AAT​GAT​GCT​CAT​CCT​CTG​CTT​GTT​CAC​TGCTC​GAG**.

After 8 h of, cells were treated with either vehicle (0.1% DMSO), 500 nM abamectin (Sigma #31732), or 5 μM abamectin in triplicate for each condition. After 48 h, the cells were harvested for reporter gene activity. A luciferase assay and β-gal assay were run on the harvested cells. The raw luciferase values were normalized to the β-gal values.

#### 2.5.2 Statistical Methods

Data from the luciferase assay was analyzed in R (R Core Team, 2020). The *lm* command from the stats package was used to build a linear model to estimate differences between inserts and identify any interactions between dose and insert. One-way ANOVA was done in R to compare across dose (0, 0.5, 5 μM abamectin) and insert (control, TT or, GG) and to test for interaction between all dose and insert combinations. Tukey’s honest significant difference test was used to compare different insert, dose pairs using *tukeyHSD* from r stats.

### 2.6 Electrophoretic Mobility Shift Assay

#### 2.6.1 Embryo Preparation

At 48 hpf embryonic chorions were mechanically removed with forceps for any unhatched embryos and three pools of 120 embryos each were collected. Embryos were anesthetized with 0.0072% tricaine methanesulfonate prior to protein extraction.

#### 2.6.2 Nuclear Protein Extraction

Manufactures protocol for tissue nuclear protein extraction was followed using ThermoFisher NE-PER nuclear and Cytoplasmic Extraction Kit (78833) and Halt Protease Inhibitor Cocktail, EDTA-free (×100) (78425) without modifications. Final nuclear extract volume was 200 μl per sample.

Nuclear protein fraction concentrations were calculated using Pierce BCA Protein Assay kit (ThermoFisher, #23225) and a Beckman DU800 spectrophotometer. Extracts ranged from 1.8 to 2.2 μg/μl.

#### 2.6.3 Oligo Design

Oligo sequences were chosen using predicted binding site sequence for the upstream region of *sox7* from the Genomatix analysis. Both sequences were design to be 26 bps long so that binding differences couldn’t be attributed to differences in length. The TT oligo contains the sequence on either side of the indel with the deletion. The TT oligo is extended by 16 bps in the 3′ direction, following the sequence in the region of interest to reach the required 26 bps.

#### 2.6.4 Oligo Preparation

Single strand oligos were synthesized by IDT, (San Diego, CA, United States) as either unlabeled or 5′ end labeled with LI-COR IRDye^®^ 700. The oligo sequences are as follows:

Labeled Sense GG version: 5′IRD700/CATGCTAAATATATATATATATATGT.

Labeled Antisense GG version: 5′IRD700/ACATATATATATATATATTTAGCATG.

Unlabeled competition GG version sense: 5′CAT​GCT​AAA​TAT​ATA​TAT​ATA​TAT​GT.

Unlabeled competition GG version antisense: 5′ACA​TAT​ATA​TAT​ATA​TAT​TTA​GCA​TG.

Unlabeled competition TT version sense: 5′CAT​GCT​AAA|TAT​ATA​TTT​GTG​CTT​TA.

Unlabeled competition TT version antisense: 5′TAA​AGC​ACA​AAT​ATA​TAT​TTA​GCA​TG.

Oligos were reconstituted in TE buffer (10 mM Tris, 1 mM EDTA, pH 8.0) and diluted to 20 μM. Sense and complimentary antisense oligos were annealed by mixing 20 μl of each paired strand, heating at 100° for 5 min followed by slowly cooling tube in heat block to room temperature overnight. Annealed IR700 oligo was then further diluted 1:200 and small aliquots were stored at −20°C. Competitor oligos stocks were kept at 20 μM equaling ×200 the labeled oligo concentration.

#### 2.6.5 Binding and Competition Assays

Odyssey EMSA Kit reagents were purchased from LI-COR Biosciences, Inc., Lincoln, NE, United States, Optimization for DNA/protein binding conditions were performed according to manufactures suggested protocol. Optimal binding buffer components were found to be as follows: 2 μl 10× Binding Buffer, 2ul 25 mM DTT/2.5% Tween20, 1 μl 1 M KCL, 1 μl 100 nM end-labeled oligo (100 fmol), 5.6 μg nuclear protein extract, water to final 20 μl volume. Optimization trials with varying concentrations of poly (dI·dC) indicated it interfered with DNA binding and was left out of subsequent runs. Reagents were mixed in microtube and incubated for 30 min at room temperature. 2 μl of ×10 Orange load dye was added immediately before loading entire sample in to wells of a BioRad Mini-Protean 5% TBE precast gel. Electrophoresis was run in ×0.5 TBE buffer at 70 V for 60–70 min.

For competition assays, unlabeled oligos were tested in a range from ×200 down to ×12.5 (20–1.25 pmol) of IR700 oligo concentration. Competitors were added to reaction mix first and incubated for 10 min to allow binding to protein before addition of labeled oligo. Reaction then incubated 20 min longer. Images were acquired on the Azure 600 Imaging System, (Azure Biosystems, Inc., Dublin, CA, United States) and image was inverted for better definition.

## 3 Results

### 3.1 Deep Reanalysis of Region Associated With Responses to Exposure

**FIGURE 2:**
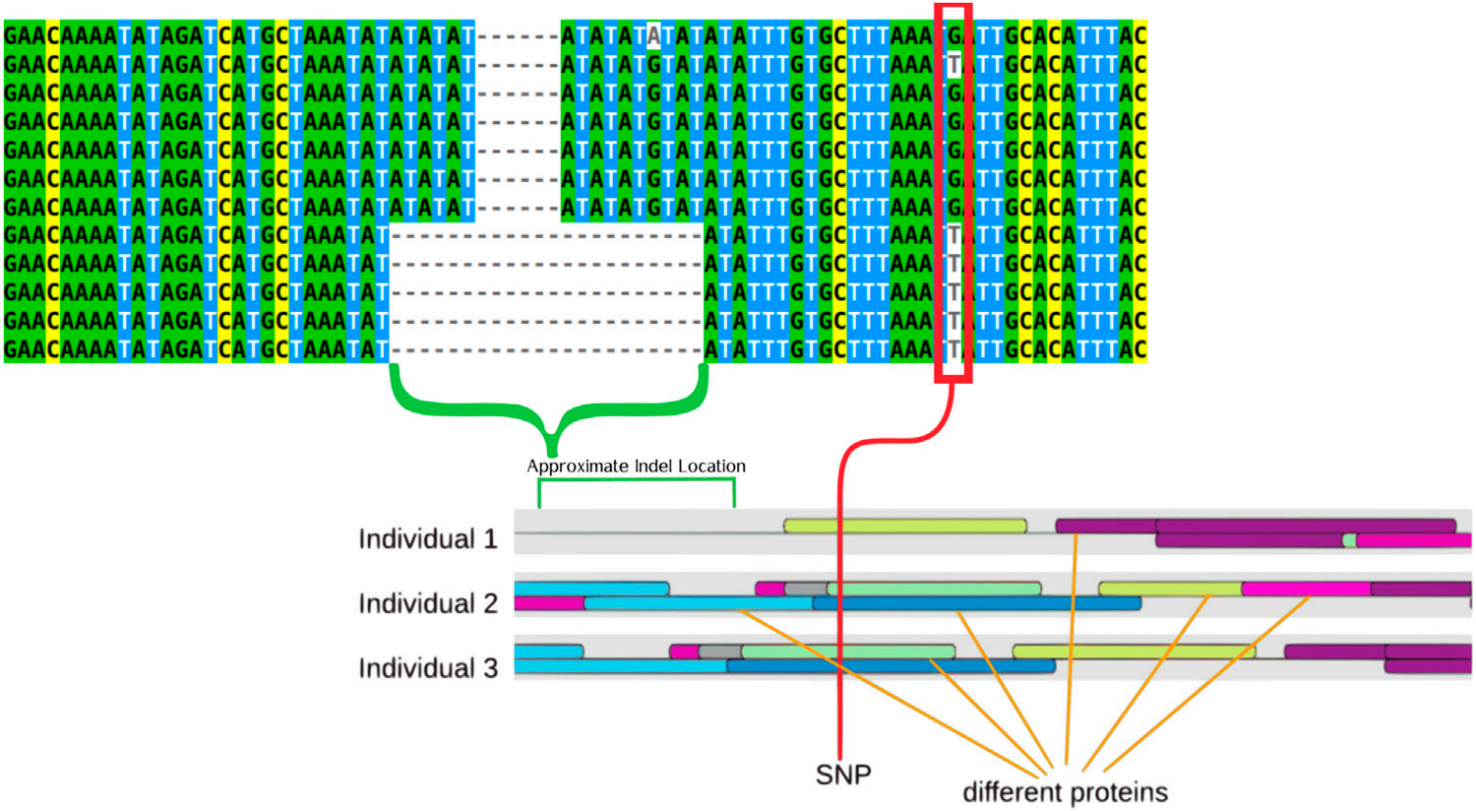
Multiple sequence alignment that displays examples of the two primary genotypes (TT and GG) the larger deletion can be seen tied to the T genotype. The indel is highlighted by a green bracket while a red rectangle highlights the SNPs. These features are also highlight on a drawing that represents the results of the transcription factor binding site prediction. The colored ovals represent different proteins, with three different representative individuals showing how protein binding is predicted differently by the software in different individuals.

Utilizing data from previous GWAS work published in Balik-Meisner et al. (2018a) and previous HTS data from Truong et al. (2014) the region identified as being associated with differences in individual susceptibility to abamectin exposure was analyzed to identify further genetic differences that may be contributing to susceptibility differences across fish (Truong et al., 2014; Balik-Meisner et al., 2018b). This analysis identified multiple SNPs and an Indel that showed significant association with these differences in susceptibility. The most significantly associated SNP as well as the indel can be seen in Figure 2. These polymorphisms represent the most common alleles in a highly variable region of the zebrafish genotype upstream of *sox7*, an important gene in development (Stovall et al., 2014).

The G/T SNP was associated with the indel in that the T allele was always paired with the deletion whereas the G allele was not. The T genotype was associated with increased susceptibility to abamectin exposure. These polymorphisms are displayed in Table 1. These polymorphisms are both upstream of *sox7* and located within a non-coding region.

**TABLE 1:** The SNP and Indel with variants listed as the reference (0) and alternate (1) alleles. Prevalence of the homozygous genotypes for both variants in affected and unaffected fish are shown in the table with the total number of fish with both genotypes at the bottom.

	Ref: G (0) Alt: T (1) Pos: 19066027		Ref: ATATATATATATATATG (0) Alt: A (1) Pos: 19065990	
	0/0	1/1	0/0	1/1
Affected	58	20	33 (30 are G/G)	16 (All T/T)
Unaffected	78	9	51 (50 are G/G)	8 (All T/T)
Total	136	29	84	24

### 3.2 *In Silico* Analysis to Identify Possible Mechanisms

#### 3.2.1 Transcription Factor Binding Site Prediction

**FIGURE 3:**
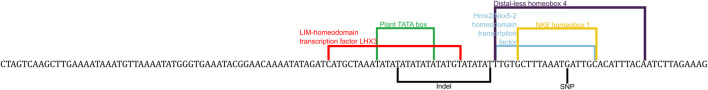
Predicted transcription factor binding sites that are in the region of interest. This sequence contains the insert and the G polymorphism of the SNP with the predicted binding sites bracketed and labelled.

One possible mechanism wherein an upstream sequence causes differential expression that leads to susceptibility differences is by acting as enhancers or promoters that recruit different transcription factors, leading to differential regulation of the downstream gene. The pathway enrichment analysis tool in the Comparative Toxicogenomics Database (CTD) shows that abamectin is enriched for effects on the signal transduction pathway which contains *sox7* (Davis et al., 2019). To test this possibility, transcription factor binding site prediction was employed using the Matbase package that is a part of the Genomatix software (Cartharius et al., 2005). Many differences in TFBSs were identified across all fish in the region upstream of *sox7*. Several of these were in the region that contains the indel and SNP. Some of these predicted sites are illustrated in Figure 3 and all predicted binding sites are present in Supplementary Table S1.

#### 3.2.2 Analysis of Predicted Transcription Factor Binding Site

To identify significant differences between TFBSs, a chi-squared test was used to compare differences between TFBSs between all of the identified polymorphisms. Two different sequences stood out as having multiple highly significant differences between predicted TFBSs. One which contained the deletion that was previously identified as well as the T allele of the G/T SNP (TT) and one which contained the G allele (GG).

**FIGURE 4:**
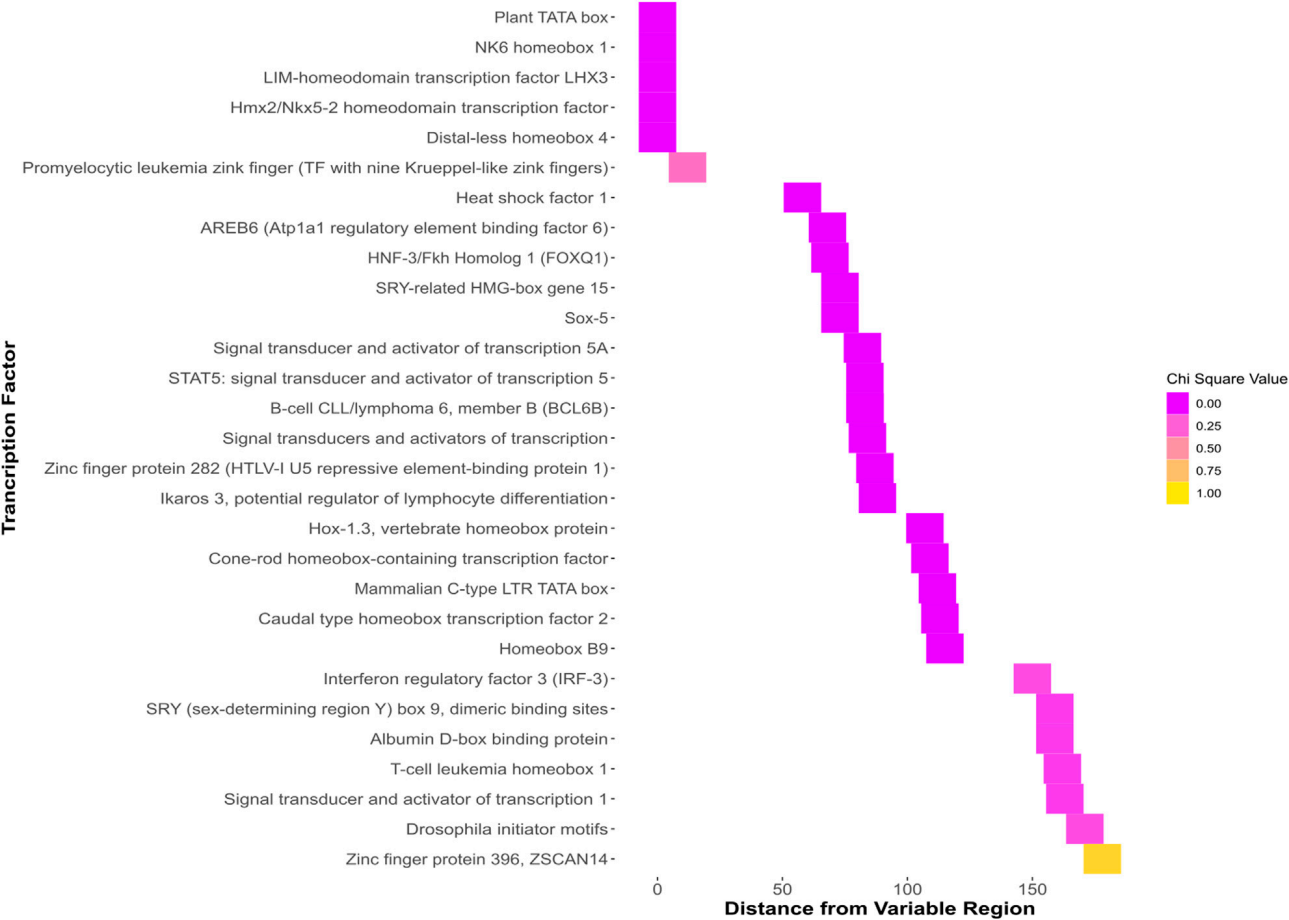
Heatmap showing all binding sites predicted to bind the GG sequence. The *y* axis shows the name of each predicted binding site. The *x* axis shows the distance of each predicted site from the region beginning with the indel and ending with the SNP. The colors, ranging from dark pink to yellow represent the chi-squared value when the predicted binding sites of the GG and TT genotypes are analyzed with a chi-squared test.

Predicted TFBSs with significant chi-squared values that were within the region of interest containing the SNP and indel (Figure 4.) were identified. Differential protein binding of these proteins *in vivo* could contribute to susceptibility differences in individuals with differing sequences at this locus.

### 3.3 Evaluating Protein Binding Between Sequences

#### 3.3.1 Electrophoretic Mobility Shift Assay Binding Assay

**FIGURE 5:**
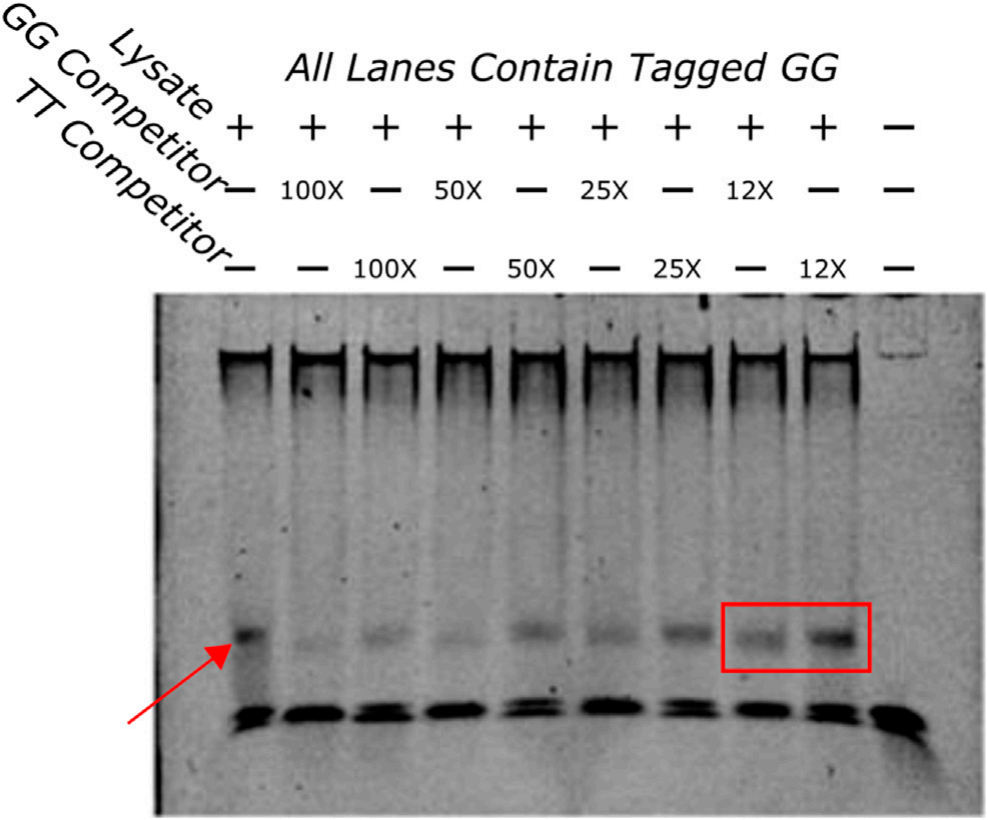
EMSA showing a competition assay done with varying concentrations of untagged GG and TT oligos. The first lane has no competitors in it and demonstrates the shift that happens when the tagged GG oligo and the protein lysate are added to the well. The band is highlighted by a red arrow. An example of the difference in bands when the GG or TT competitors are added is highlighted with a red box. The GG competitor produces a much lighter band, indicating that the GG sequence has more affinity for the protein that is being bound than the TT sequence.

EMSA was done to establish protein-DNA interaction of the GG sequence *in vitro*. The first assay evaluated if the GG sequence was able to bind protein in zebrafish protein lysate. As seen in Figure 5 the sequence was shown to bind to a protein in the lysate.

#### 3.3.2 Electrophoretic Mobility Shift Assay Competition Assay

To determine if the sequences had different binding affinities to the same protein a competition binding assay was done with a tagged GG oligo and an excess of untagged competitor TT or GG oligos at varying concentrations. If the competitors bound the same proteins with different affinities the untagged TT would pull away protein from the tagged GG sequence but with less efficiency than the untagged GG oligo. This would appear as a fainter band in wells that contained the untagged GG oligo compared to wells that contained the untagged TT oligo indicating that the TT sequence bound with less affinity than the GG sequence. Figure 5 indicates that this is the case. A fainter band can be identified clearly in several GG competition lanes when compared to the same concentrations of the TT competition wells. These results indicate that these two sequences bind differentially to the same protein.

### 3.4 Investigating the Regulatory Activity of Different Sequences

#### 3.4.1 Luciferase Assay

**FIGURE 6:**
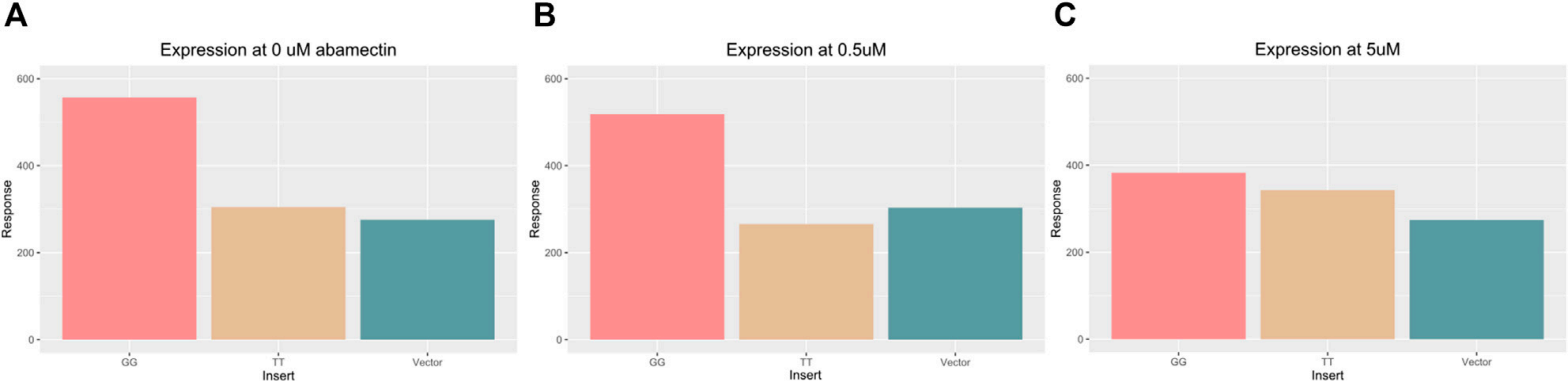
Bar chart displaying the average luciferase activity across three different vector inserts (GG, TT, and control) at 0 μM **(A)** and 0.5 μM **(A)** and 5 μM abamectin **(C)**. The GG vector (left most) has higher expression in all three environments. The TT vector (middle) and control vector (right most) look similar and show lower expression except in response to the highest level of abamectin expression.

The substantial predicted binding site differences between the GG and TT sequences, and the differential protein binding that was identified indicate that this locus may play an important role in *sox7* expression. To test if these polymorphisms alone, along with their different binding behavior could lead to expression differences, TT and GG were inserted into luciferase vectors which were transfected into MCF7 cells. Figure 6 shows luciferase expression in the cells across the three doses.

#### 3.4.2 Statistical Analysis

Significant differences were found in luciferase expression across inserts in an ANOVA (Df = 2, F = 41.4, *p* = 5.1e−8). When compared using Tukey’s test, cells with the TT or GG insert showed significant differences in luciferase expression (*p* adjusted = 4.6e−4). Cells with the TT or GG insert that were exposed to 0.5 μM of abamectin also showed significant expression differences as well (*p* adjusted = 4.7e−4). There was also a significant difference between the GG cells and the control vector transfected cells when there was no exposure (*p* adjusted = 1.3e−04) and when they were exposed to 0.5 μM of abamectin (*p* adjusted = 2.7e−03). The TT cells and control cells behaved similarly with regards to luciferase expression with no significant differences identified between them at either dose. There were no observed changes in expression in the cells with the same vector at different doses. While not visually apparent across the first two doses, abamectin dose had a significant affect on *sox7* expression when comparing across all three doses in a linear regression (*t* = −4.29, *p* = 0.003).

## 4 Discussion

The results of this study reinforce the hypothesis that the sequences upstream of *sox7* containing the SNP and Indel that were profiled in this study act as regulators of the *sox7* gene, where regulatory differences caused by the two genotypes lead to differences in susceptibility to chemical exposure. The position of the variants upstream of Sox7 in a non-coding region, combined with the fact that two specific variants were highly associated with different outcomes after exposure points to the possibility that these sequences play a regulatory role in the expression of *sox7* as promoters, enhancers, and/or repressors. One mechanism that could explain the substantial differences in the effects of exposure and the expression of *sox7* is that these variants, in their role as regulators, bind different transcription factors. These binding differences then instigate differential regulation of Sox7 expression which leads to discrepancies in susceptibility to developmental defects brought on by chemical exposure.

*In silico* analysis was done to look at possible TFBS differences between the two sequences which could bring about the observed expression differences. This analysis predicted that many of the TFBSs differed at the deletion and SNP loci. This led to the conclusion that these two sequences likely bind some factor with differing affinity, resulting in the observed expression differences between individuals with different genotypes. These results were used to guide further wet-lab experimentation.

Using EMSA, the ability of these sequences to bind protein was investigated. The results of the initial EMSA looking at the ability of the GG sequences to bind protein *in vitro* demonstrated that it bound a protein that is present during zebrafish development. A second EMSA, looking at competition between the two sequences, illustrated that there were pronounced differences in binding affinity between the two variants.

Binding differences between two sequences does not necessarily explain the expression differences that were associated with different developmental outcomes. A luciferase assay clearly displayed significant expression differences that were precipitated by the two different genotypes when inserted, with a minimal promoter, into human cells. This demonstrates that even without the longer promoter sequence; the SNP and indel were sufficient to change *sox7* expression.

The mechanism of differential susceptibility identified in this study is as follows: The differences in the hypervariable region upstream of *sox7* lead to differences in the suite of transcription factors that bind the DNA to affect *sox7* expression. This, in turn, causes differential expression of the important developmental factor Sox7. These expression differences during an organism’s development contribute to susceptibility differences between organisms when exposed to abamectin. While the next steps in the adverse outcome pathway are subject to additional studies, we hypothesize that these expression differences might be connected to a developmental pathway involving *sox7* that is disrupted by abamectin without being able to compensate by expressing more protein.

Sox7 plays a crucial role in development in humans and fish alike (Stovall et al., 2014). Sox7 plays an especially important role in the development of hematopoietic pathways and vasculogenesis in many vertebrates including zebrafish, humans, and rodents (Pendeville et al., 2008; Francois et al., 2010; Carroll and North, 2014; Lomelí and Castillo-Castellanos, 2020). Differential expression of *sox7* could, therefore, cause susceptibility differences to chemical exposure, particularly during development. Sox7 downregulation has also been linked to breast cancer in humans (Stovall et al., 2014). Changes in the regulatory sequence upstream of SOX7 may be one factor responsible for these changes in expression related to breast cancer.

This study identified a hypervariable region upstream of the zebrafish *sox7* gene. Analysis of the region upstream of SOX7 in the Ensembl human genome assembly (GRCh38.p13) shows a known promoter and enhancer region on chromosome 8 from base pairs 10730506 to 10736999 (Chromosome 8: 10,730,506–10,736,999, 8). The genome aggregation database (GnomAD) contains over 700 short variants (SNPs and Indels) that have been identified in this region but have not been investigated for phenotypic effects nor ties to susceptibility differences after chemical exposure (8-10730506-10736999 | gnomAD v2.1.1 | gnomAD). Non-coding regions are understudied, and there is little information on the evolutionary conservation of the region upstream of SOX7 between zebrafish and humans. Studies on the protein itself, though, have shown that there is 78% identity between the human and the zebrafish protein (Francois et al., 2010). This points to similarities being conserved across humans and zebrafish in this region that could point to important implications regarding human susceptibility to abamectin and other compounds that affect development through interactions with SOX7. Further investigation could use human data on exposure to abamectin and other related compounds (see concluding paragraph below) to uncover possible GxE and susceptibility differences that were implicated in this study and flagged in the initial high-throughput screening. The broader implications of this study are that a high-throughput screening method in a diverse population can successfully identify compounds that elicit GxE causing observable effects.

Zebrafish are the only vertebrate model that is currently compatible with high-throughput methods and shares a large degree of evolutionary conservation with humans (Garcia et al., 2016). Moreover, the HTS experiments described here cover the phylotypic stage, wherein vertebrate development across taxa are most similar, making developmental comparisons across vertebrates especially useful (Garcia et al., 2016). Zebrafish have proven especially useful in the study of human fetal development, vasculogenesis, hematopoiesis, brain development, thyroid function, and the hypothalamic-pituitary-thyroid axis (Pendeville et al., 2008, Liu et al., 2013; Carroll and North, 2014; Duboc et al., 2015; Garcia et al., 2016; Vancamp and Darras, 2018; Lomelí and Castillo-Castellanos, 2020). Thus, an HTS-compatible zebrafish model is adaptable to identifying GxE mechanisms behind susceptibility differences to chemical exposure that may be of direct human relevance. In future studies of additional exposures, the discovery path described here would necessarily be tailored or branched according to empirical results. This flexible method lays a foundation for the rapid discovery of chemicals that participate in GxE and the unraveling of their mechanisms.

Despite the promise and functionality of this high-throughput discovery method, the model that is being utilized does have drawbacks. The speed at which the screening is done imposes constraints on the breadth and depth of the analysis. As with any model, zebrafish are not a sufficient substitute for human studies. While they serve as important models that share a great deal of similarity with humans, there are many mechanistic and genetic differences between humans and zebrafish. Examples of studies aiming to understand these differences have documented how hematopoiesis occurs in a different organ, primitive blood cells are only unipotent, and there are some differences in signaling during development (Carroll and North, 2014; Duboc et al., 2015; Lomelí and Castillo-Castellanos, 2020).

Considering these strengths and limitations, we propose zebrafish HTS as an approach to begin addressing the paucity of information on GxE causing individual susceptibility differences to the tens of thousands of chemicals in the growing exposome. Analysis at this scale could rapidly screen for signals that require further investigation. Future studies would then be able to find mechanisms of GxE involving compounds of public health interest by following the information gleaned from HTS. Moreover, while identification of new and underinvestigated compounds that may be tied to differential susceptibility presents on ongoing challenge, the same critical question must be asked of otherwise well-studied compounds. In this manner, human health can be improved by new knowledge regarding existing, new, and well-studied compounds. Abamectin acts as a case study in the importance of gaining a better understanding of well-studied chemicals like the mectin family of compounds, where new exposures and usage patterns can introduce population health concerns. As illustration, consider data from the Centers for Disease Control showing that prescriptions for ivermectin for humans increased more than ten-fold, from 3,600 to 39,000, in 2020 amidst the COVID-19 pandemic (Health Alert Network (HAN), 2021). This method uses high-dimensional HTS data are interrogated for signals of differential susceptibility, also addresses some of the problems related to the assessment of differential individual susceptibility to chemical exposure. Namely, problems with sample sizes of sufficient power, mechanistic investigation, environmental variables, and the relationship between exposure and effect. Combining novel *in silico* and HT methods with existing *in vitro* and *in vivo* methods, this approach could lead to a new understanding of GxE and differential susceptibility that changes the current paradigm for parsing out GxE in diverse populations. Understanding GxE and the factors that contribute to susceptibility can lead to better risk assessment, a better grasp on the effects of a chemical, more effective regulation to improve human health, and an increasing knowledge of adverse outcome pathways.

## Data Availability

The datasets presented in this study can be found in online repositories. The names of the repository/repositories and accession number(s) can be found below: https://www.ncbi.nlm.nih.gov/, PRJNA482650.

## Supplementary Material

Supplementary Table S1 Predicted transcription factor binding sites in the region upstream of sox7 in T5D zebrafish.
