# Supplementary material for: Leveraging a High-Throughput Screening Method to Identify Mechanisms of Individual Susceptibility Differences in a Genetically Diverse Zebrafish Model
Source: Front Toxicol. 2022 Apr 29;4:846221. doi: 10.3389/ftox.2022.846221 (PMC9098949; doi:10.3389/ftox.2022.846221)
Supplement: Supplementary file 1 [file DataSheet1.DOCX]

| Transcription Factor | Description |
| --- | --- |
| V$CDX1.01 | Intestine specific homeodomain factor CDX-1 |
| V$HMGA.01 | HMGA family of architectural transcription factors (HMGA1, HMGA2) |
| V$NKX61.02 | NK6 homeobox 1 |
| V$PIT1.03 | GHF-1 pituitary specific pou domain transcription factor |
| V$BRN2.01 | Brn-2, POU-III protein class |
| V$LHX3.02 | LIM-homeodomain transcription factor LHX3 |
| V$ISL2.01 | ISL LIM homeobox 2 |
| V$SALL1.01 | Spalt-like transcription factor 1 |
| V$TEF.01 | Thyrotrophic embryonic factor |
| V$HMX2.03 | Hmx2/Nkx5-2 homeodomain transcription factor |
| V$HOXB9.01 | Abd-B-like homeodomain protein Hoxb-9 |
| V$FOXP1_ES.01 | Alternative splicing variant of FOXP1, activated in ESCs |
| V$MSX2.01 | Muscle segment homeo box 2, homologue of Drosophila (HOX 8) |
| V$BCL6.04 | B-cell CLL/lymphoma 6, member B (BCL6B) |
| V$CEBPE.02 | CCAAT/enhancer binding protein (C/EBP), epsilon |
| V$AREB6.04 | AREB6 (Atp1a1 regulatory element binding factor 6) |
| V$GATA1.01 | GATA-binding factor 1 |
| V$SMARCA3.01 | SWI/SNF related, matrix associated, actin dependent regulator of chromatin, subfamily a, member 3 |
| V$STAT5.01 | STAT5: signal transducer and activator of transcription 5 |
| V$NMP4.01 | NMP4 (nuclear matrix protein 4) / CIZ (Cas-interacting zinc finger protein) |
| V$GSH2.01 | Homeodomain transcription factor Gsh-2 |
| V$STAT5A.01 | Signal transducer and activator of transcription 5A |
| V$CEBPB.02 | CCAAT/enhancer binding protein beta |
| V$YY1.02 | Yin and Yang 1 repressor sites |
| V$CDX2.03 | Caudal type homeobox transcription factor 2 |
| V$CRX.03 | Cone-rod homeobox-containing transcription factor |
| V$SATB1.01 | Special AT-rich sequence-binding protein 1, predominantly expressed in thymocytes, binds to matrix attachment regions (MARs) |
| V$PAX2.02 | Paired box protein 2 |
| V$IRF3.01 | Interferon regulatory factor 3 (IRF-3) |
| O$VTATA.01 | Cellular and viral TATA box elements |
| O$DINR.01 | Drosophila initiator motifs |
| V$MYT1L.01 | Myelin transcription factor 1-like, neuronal C2HC zinc finger factor 1 |
| V$HAS.01 | HIF-1 ancillary sequence |
| V$OCT2.02 | Octamer-binding transcription factor-2, POU class 2 homeobox 2 (POU2F2) |
| V$STAT.01 | Signal transducers and activators of transcription |
| O$VTATA.02 | Mammalian C-type LTR TATA box |
| V$HOXD10.01 | Homeobox D10 |
| V$PLZF.02 | Promyelocytic leukemia zink finger (TF with nine Krueppel-like zink fingers) |
| V$MESP1_2.01 | Mesoderm posterior 1 and 2 |
| O$PTATA.02 | Plant TATA box |
| V$HOXB9.02 | Homeobox B9 |
| V$HOXD13.01 | Homeobox D13 / Hox-4I |
| V$FAST1.02 | Forkhead box H1 (Foxh1) |
| V$LMX1B.01 | LIM-homeodomain transcription factor |
| V$OCT3_4.02 | POU domain, class 5, transcription factor 1 |
| V$BCL6.03 | B-cell CLL/lymphoma 6, member B (BCL6B) |
| V$TST1.01 | POU-factor Tst-1/Oct-6 |
| V$BRIGHT.01 | Bright, B cell regulator of IgH transcription |
| V$PCE1.01 | Photoreceptor conserved element 1 |
| V$CART1.01 | Cart-1 (cartilage homeoprotein 1) |
| V$PROP1.02 | Prophet of Pit 1, PROP paired-like homeobox 1, dimeric binding site |
| V$IRF4.03 | Interferon regulatory factor 4 |
| V$HOXC9.02 | Member of the vertebrate HOX - cluster of homeobox factors |
| V$XBP1.01 | X-box-binding protein 1 |
| V$GATA2.02 | GATA-binding factor 2 |
| V$IK3.01 | Ikaros 3, potential regulator of lymphocyte differentiation |
| V$MSX1.01 | Muscle-segment homeobox 1, msh homeobox 1 |
| O$SPT15.01 | TATA-binding protein, general transcription factor that interacts with other factors to form the preinitiation complex at promoters |
| V$BRN4.01 | POU domain transcription factor brain 4 |
| V$OC2.01 | CUT-homeodomain transcription factor Onecut-2 |
| V$CEBPA.02 | CCAAT/enhancer binding protein alpha |
| V$TLX1.01 | T-cell leukemia homeobox 1 |
| V$BRN3.02 | Brn-3, POU-IV protein class |
| V$FHXB.01 | Fork head homologous X binds DNA with a dual sequence specificity (FHXA and FHXB) |
| V$XFD2.01 | Xenopus fork head domain factor 2 (FoxI1a) |
| V$DLX4.01 | Distal-less homeobox 4 |
| V$SOX15.01 | SRY-related HMG-box gene 15 |
| V$NUR77.01 | Nuclear hormone receptor NUR77 (NR4A1) |
| V$SPIB.01 | Spi-B transcription factor (Spi-1/PU.1 related) |
| V$PHOX2.01 | Phox2a (ARIX) and Phox2b |
| O$PTATA.01 | Plant TATA box |
| V$GCM1.03 | Glial cells missing homolog 1 (secondary DNA binding preference) |
| V$SRY.02 | Sex determining region Y |
| V$PAX2.01 | Zebrafish PAX2 paired domain protein |
| V$CLOX.01 | Cut-like homeodomain protein |
| V$HNF6.02 | Liver enriched Cut - Homeodomain transcription factor HNF6 (ONECUT1) |
| V$PAX6.02 | PAX6 paired domain and homeodomain are required for binding to this site |
| V$SOX5.01 | Sox-5 |
| V$PAX6.01 | Pax-6 paired domain binding site |
| V$FAST1.01 | FAST-1 SMAD interacting protein |
| V$EN1.02 | Engrailed homeobox 1 |
| V$ESX1.01 | ESX homeobox 1 |
| V$ZID.01 | Zinc finger with interaction domain |
| V$GSH1.01 | Homeobox transcription factor Gsh-1 |
| V$HFH1.01 | HNF-3/Fkh Homolog 1 (FOXQ1) |
| V$OCT1.03 | Octamer-binding transcription factor-1, POU class 2 homeobox 1 (POU2F1) |
| V$PARAXIS.01 | Paraxis (TCF15), member of the Twist subfamily of Class B bHLH factors, forms heterodimers with E12 |
| V$HSF1.01 | Heat shock factor 1 |
| V$PROX1.01 | Prospero homeobox protein 1, dimeric binding site |
| V$HOXA3.01 | Homeobox A3 |
| V$NKX63.01 | NK6 homeobox 3 |
| V$DBP.01 | Albumin D-box binding protein |
| V$HOXA9.02 | Homeobox A9 / Hox1-gamma |
| O$MTATA.01 | Muscle TATA box |
| V$IRX6.01 | Iroquois homeobox 6 |
| V$HMBOX.01 | Homeobox containing 1 |
| V$LHX2.01 | LIM homeobox 2 |
| V$PAX6.04 | PAX6 paired domain binding site |
| V$HBP1.02 | HMG box-containing protein 1 |
| V$OCT1.06 | Octamer-binding factor 1 |
| V$HOX1-3.01 | Hox-1.3, vertebrate homeobox protein |
| V$RORA2.01 | RAR-related orphan receptor alpha2 |
| V$SL1.01 | Member of the RSRF (related to serum response factor) protein family from Xenopus laevis |
| V$PAX4.02 | Paired box 4, homeodomain binding site |
| V$HOXB4.01 | Homeobox B4 / Hox-2phi |
| V$HOXB3.01 | Homeobox B3 / Hox 2-gamma |
| V$OSNT.01 | Composed binding site for Oct4, Sox2, Nanog, Tcf3 (Tcf7l1) and Sall4b in pluripotent cells |
| V$PRDM1.01 | PRDI binding factor 1 |
| V$PAX7.01 | Paired box 7 homeodomain-binding motif |
| V$ZNF396.01 | Zinc finger protein 396, ZSCAN14 |
| V$E4BP4.01 | E4BP4, bZIP domain, transcriptional repressor |
| V$OCT1.04 | Octamer-binding factor 1 |
| V$POU3F3.01 | POU class 3 homeobox 3 (POU3F3), OTF8 |
| V$SOX10.03 | SRY (sex determining region Y)-box 10, dimeric binding sites |
| V$PIT1.02 | POU domain, class 1, transcription factor 1 (POU1F1) / Pituitary transcription factor-1 |
| V$PBX_HOXA9.01 | PBX - HOXA9 binding site |
| V$P53.05 | Tumor suppressor p53 |
| V$OCT3_4.01 | POU domain, class 5, transcription factor 1 |
| V$DMRT4.01 | Doublesex and mab-3 related transcription factor 4 |
| V$BCL6.02 | POZ/zinc finger protein, transcriptional repressor, translocations observed in diffuse large cell lymphoma |
| V$BRN4.02 | POU class 3 homeobox 4 (POU3F4) |
| V$DMRT2.01 | Doublesex and mab-3 related transcription factor 2 |
| V$DMRT3.01 | Doublesex and mab-3 related transcription factor 3 |
| V$ATBF1.01 | AT-binding transcription factor 1 |
| V$BRN3.01 | Brn-3, POU-IV protein class |
| V$SOX1.04 | SRY (sex determining region Y)-box 1, dimeric binding sites |
| V$EVI1.03 | Ecotropic viral integration site 1 encoded factor, amino-terminal zinc finger domain |
| V$BRN5.03 | Brn-5, POU-VI protein class (also known as emb and CNS-1) |
| V$ZFP652.01 | Zinc finger protein 652 (ZNF652) |
| V$EN1.01 | Homeobox protein engrailed (en-1) |
| V$BRN5.01 | Brn-5, POU-VI protein class (also known as emb and CNS-1) |
| V$POU6F2.01 | Retina-derived POU-domain factor-1, dimeric binding site |
| V$SOX1.01 | SRY (sex determining region Y)-box 1, dimeric binding sites |
| V$STAT1.01 | Signal transducer and activator of transcription 1 |
| V$MEF2.01 | Myocyte-specific enhancer factor 2 |
| V$SOX9.08 | SRY (sex-determining region Y) box 9, dimeric binding sites |
| V$PSE.01 | Proximal sequence element (PSE) of RNA polymerase II-transcribed snRNA genes |
| V$ZNF282.01 | Zinc finger protein 282 (HTLV-I U5 repressive element-binding protein 1) |
| V$GZF1.01 | GDNF-inducible zinc finger protein 1 (ZNF336) |
| V$EVI1.01 | Ecotropic viral integration site 1 encoded factor, amino-terminal zinc finger domain |
| V$E2F.01 | E2F, involved in cell cycle regulation, interacts with Rb p107 protein |
| V$AIRE.02 | Autoimmune regulator |

**Supplemental table** 1. Predicted transcription factor binding sites in the region upstream of *sox7* in T5D zebrafish.
